# Thematic and Spatial Resolutions Affect Model-Based Predictions of Tree Species Distribution

**DOI:** 10.1371/journal.pone.0067889

**Published:** 2013-07-04

**Authors:** Yu Liang, Hong S. He, Jacob S. Fraser, ZhiWei Wu

**Affiliations:** 1 State Key Laboratory of Forest and Soil Ecology, Institute of Applied Ecology, Chinese Academy of Sciences, Shenyang, Liaoning, China; 2 School of Natural Resources, University of Missouri, Columbia, Missouri, United States of America; DOE Pacific Northwest National Laboratory, United States of America

## Abstract

Subjective decisions of thematic and spatial resolutions in characterizing environmental heterogeneity may affect the characterizations of spatial pattern and the simulation of occurrence and rate of ecological processes, and in turn, model-based tree species distribution. Thus, this study quantified the importance of thematic and spatial resolutions, and their interaction in predictions of tree species distribution (quantified by species abundance). We investigated how model-predicted species abundances changed and whether tree species with different ecological traits (e.g., seed dispersal distance, competitive capacity) had different responses to varying thematic and spatial resolutions. We used the LANDIS forest landscape model to predict tree species distribution at the landscape scale and designed a series of scenarios with different thematic (different numbers of land types) and spatial resolutions combinations, and then statistically examined the differences of species abundance among these scenarios. Results showed that both thematic and spatial resolutions affected model-based predictions of species distribution, but thematic resolution had a greater effect. Species ecological traits affected the predictions. For species with moderate dispersal distance and relatively abundant seed sources, predicted abundance increased as thematic resolution increased. However, for species with long seeding distance or high shade tolerance, thematic resolution had an inverse effect on predicted abundance. When seed sources and dispersal distance were not limiting, the predicted species abundance increased with spatial resolution and vice versa. Results from this study may provide insights into the choice of thematic and spatial resolutions for model-based predictions of tree species distribution.

## Introduction

Concerns about global climate change, habitat fragmentation, and biodiversity loss have increasingly stimulated researchers to predict vegetation dynamics at broad spatial scales [1]–[3]. The primary tools for predicting broad-scale forest vegetation dynamics include niche models [4], [5], process models [6], [7], and forest landscape models [8]. All of these models need to account for the effects of physical environment or environmental heterogeneity in the modelling framework. Thus, characterizing environmental heterogeneity is fundamental for model-based predictions of broad-scale vegetation dynamics.

Environmental heterogeneity is characterized by the complexity in composition and configuration of a system property (e.g., land type) [9]–[11]. Compositional heterogeneity is characterized by the number of classes that describe environmental heterogeneity and the proportional area of each class in the study landscape. These classes are often known as land types or ecoregions depending on study scales (hereafter called land type). A land type corresponds to multiple spatial units (hereafter called land type unit) within which physical environments are assumed to be uniform. Configurational heterogeneity includes spatial arrangement of the land type units, their geometric shapes, contrast between neighbouring units, and connectivity among units of the same land type [12]. Landscapes with more land types or more complex spatial patterns are considered more heterogeneous [13], [14].

Spatial input to a niche, process, or forest landscape model is usually derived from classified remote sensing imagery and other GIS datasets (e.g., soil and land cover). The thematic resolution (e.g., the number of land types) and spatial resolution (grain size) for these spatial inputs is often determined by the availability of the datasets or subjectively. These subjective decisions may result in different characterizations of environmental heterogeneity. Previous studies showed that changing spatial resolution may affect characterizations of configurational heterogeneity [15]–[17], while changing thematic resolution may result in different characterizations of both compositional and configurational heterogeneity [18]–[20]. The general response of spatial patterns, directly related to characterization of environmental heterogeneity, to changing spatial resolution may resemble that of changing thematic resolution. For example, decreasing spatial resolution results in a decrease in the number of land type units, while decreasing thematic resolution leads to a decrease in the number of land types due to aggregation, which also results in reduction in the number of land type units [15], [19], [21]. The details, however, may differ significantly in spatial aggregation: decreasing spatial resolution always leads to aggregating neighbouring grid cells (following the majority rule in many cases), whereas decreasing thematic resolution results in combining cells of similar land types that may be far apart [19].

While the effects of thematic and spatial resolutions on spatial pattern are fairly well understood, effects of both resolutions on model-based predictions of species distribution have received little attention. Species establishment in a land type depends on the presence of suitable habitat, as well as the ability of the species to reach the land type (e.g., seed dispersal). Once established, the development of a tree species depends on local system dynamics, such as interspecies competition and variations in the environment over time [22]. Therefore, the fate of the species is ultimately part of the forest dynamic that is influenced by the number of habitats present and by the rates of ecological processes such as seed dispersal, competition, and migration or death. It has been shown that derivation of habitat number as well as the simulation of occurrence and rate of ecological processes are affected by thematic and spatial resolutions in characterizing environmental heterogeneity [17], [23], [24]. Therefore, both resolutions are expected to affect model-based tree species distribution, with effects magnified over time [13], [25].

Since thematic and spatial resolutions are chosen separately, it is necessary to investigate the effects of each accordingly. From an ecological perspective, increasing thematic resolution, which identifies more land types, may result in greater number of suitable habitat types [26], allowing rare species to establish and persist. Decreasing spatial resolution may impair the simulation of seed dispersal since when a cell size is larger than the effective seeding distance, as seed may fail to disperse outside the cell [25]. From a modelling perspective, increasing either thematic or spatial resolution leads to detailed characterization of spatial patterns, and consequently challenges in maintaining model accuracy [13], [25]; decreasing both resolutions can average out some chaotic behaviours at the expense of losing spatial details, and consequently improve model predictability [13], [27]. Therefore, it is important to know which, if either, resolution has a dominant effect on predictions for maximizing the effectiveness of the model in balancing modelling accuracy and model predictability.

The objective of this study is to investigate the effects of thematic and spatial resolutions in characterizing environmental heterogeneity on predictions of tree species distribution at the landscape scale (quantified by species abundance). Specifically, we (a) investigated how species abundances change with varying thematic and spatial resolutions, respectively, (b) analysed whether tree species with different ecological/biological traits (e.g., seed dispersal distance, competitive capacity) have different responses to varying thematic and spatial resolutions, and (c) evaluated the relative importance of thematic and spatial resolutions, and their interaction in predictions of species distribution. We designed a series of scenarios with different combinations of thematic (different numbers of land types) and spatial resolutions, and then statistically examined the differences of response variables (species abundance) among these scenarios.

## Approach and Methods

### 2.1 Study area

**Figure 1:**
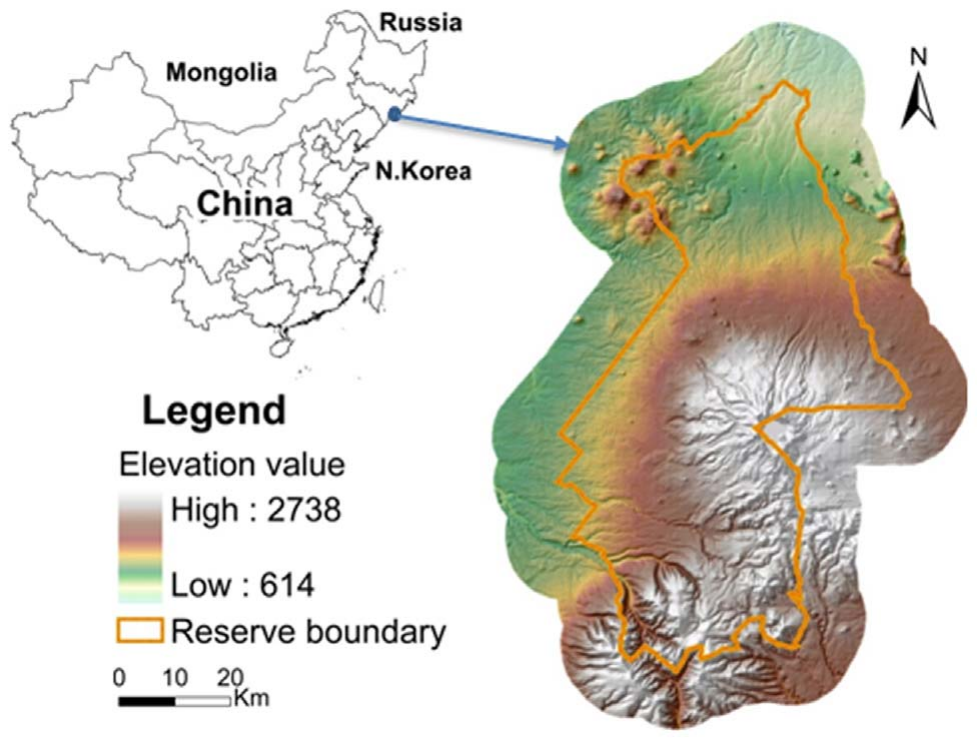
Geographic site of the study area.

Our study area (4.1×10^5^ ha) consisted of the Changbai Mountain National Natural Reserve (CMNNR) and the 8 km surrounding area at 41°62′–42°49′ N, 127°59′–128°38′ E (Fig. 1). CMNNR contains the highest mountain in northeastern China and protects one of the largest natural temperate forests in the world [28]. Main tree species include Korean pine (*Pinus koraiensis* Siebold & Zucc.), jezo spruce (*Picea jezoensis* Siebold & Zucc.), Manchurian fir (*Abies nephrolepis* [Trautv.] Maxim), Olga Bay larch (*Larix olgensis* A. Henry), Asian white birch (*Betula platyphylla* Suk), Mongolian oak (*Quercus mongolica* [Fisch] Ledeb.), and mountain birch (*Betula ermanii* Cham.). Korean pine, fir and spruce are shade-tolerant species, while larch, white birch, oak and mountain birch are relative shade-intolerant species. The seeds of Korean pine are so large that they cannot disperse by wind, instead relying on gravity and animals. Thus, Korean pine has much shorter maximum dispersal distances. The dispersal modes of fir and oak are similar to that of Korean pine. In contrast, white birch have a wide seed dispersal range.

Elevation and aspect are the most important factors that cause environmental heterogeneity in the Changbai Mountains [29]. Elevation governs broad-scale vegetation distribution patterns, which are reflected by distinct forest types corresponding to elevation gradients in our study area [30]. These forest types compose four major vertical/elevation forest zones [31]. The hardwood forest zone (lower than 750 m elevation) is mainly composed of white birch, Korean aspen (*Populus davidiana* Dode), maple (*Acer mono* Maxim), and elm (*Ulmus propinqua* Koidz.). The mixed Korean pine hardwood forest zone (750–1100 m) includes Korean pine, oak, basswood (*Tilia amurensis* Rupr.), Manchurian ash (*Fraxinus mandschurica* Rupr.), maple, and elm. The spruce–fir forest zone (1000–1700 m) is mainly composed of spruce and fir. The subalpine forest zone (1700–2000 m) is dominated by mountain birch and larch. Aspect governs fine-scale species composition by redistributing humidity and temperature in the environment [32]. Aspect can be divided into two classes (sunny slope and shady slope) and four classes (north, south, west, and east slope). Thus, land types of our study area can be characterized at three levels: 4 (4 elevation zones), 8 (4 elevation zones ×2 classes of aspects), or 16 (4 elevation zones ×4 classes of aspects) land types.

### 2.2 Landscape-scale predictions of tree species distribution

**Figure 2:**
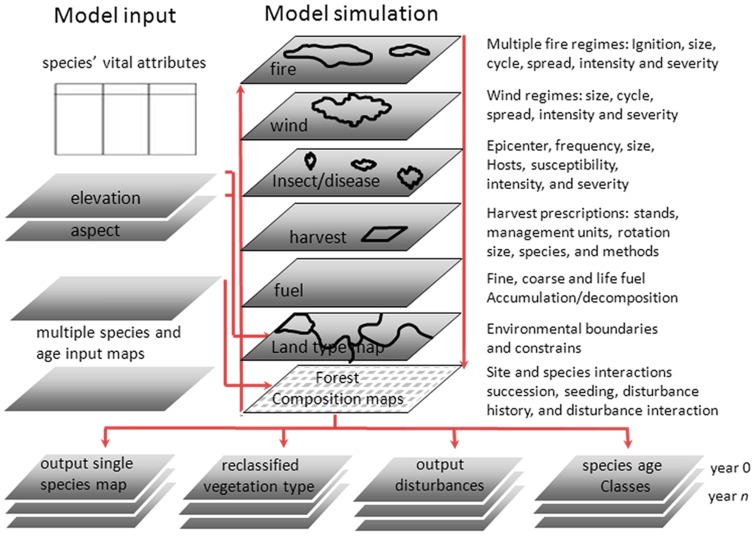
The operational design of LANDIS. The LANDIS model can be represented conceptually as a repeating cycle of processes that operate on the initial input map and subsequent time steps. Succession occurs within a cell based on species' vital attributes such as shade tolerance and longevity.

LANDIS simulates forest succession and landscape processes (e.g., seed dispersal) using spatially interactive cell-based landscapes [8], which predict landscape-scale tree species distribution under climate warming (quantified by species abundance) in this study. The parameters of LANDIS mainly included two spatial data layers (raster maps for land type and forest composition), in addition to other nonspatial parameters (e.g., species' vital attributes) (Fig. 2).

In LANDIS, a heterogeneous landscape can be delineated into various land types based on characterizations of environmental heterogeneity. These land types were derived from Digital Elevation Model, downloaded from the website of the CGIAR Consortium for Spatial Information (CGIAR-CSI, http://srtm.csi.cgiar.org/SELECTI-ON/inputCoord.asp). Within each land type, response of species to warming climate was quantified by species establishment probability under climate warming [33]. This establishment probability can be derived from an ecosystem process model, LINKAGES [34]. LINKAGES integrates environmental variables such as warming climate (monthly temperature and precipitation, downloaded from http://www.climate-wizard.org/) and soil (C, N and water, derived from soil database of China) with ecological processes (competition, succession, and water and nutrient cycling) and outputs individual species biomass. Because a larger biomass for species at the same age represented greater species suitability to the land type, biomass was used to quantify species suitability to the land type in the form of species establishment probability (SEP). Biomass for each land type under warming climate was converted to SEP for the corresponding land type under warming climate using the following equations [33].
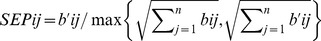
(1)Where 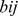 and 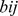 are the biomass of species *i* on land type *j* under current and warming climate, respectively. SEP for a given species may be homogeneous within each land type but vary from one land type to another [33], [35].

The second spatial input needed to run LANDIS is a forest composition map. The forest composition map sets the initial conditions for LANDIS by describing individual species–age class distribution in each raster cell for the study area and such information was derived by integrating classified remote sensing imagery and field inventory data [30], [35]. The resolution of the forest composition map in this study was 30 m largely because of the Landsat Thematic Mapper's resolution.

In LANDIS, succession and dispersal are driven by species' vital attributes. Seed dispersal probability is modeled using an exponential distribution where each species has an effective and maximum dispersal distance that controls seed distribution [36]. A seed has a higher probability of reaching a site within the species effective seeding distance than beyond this distance [36]. When a seed successfully arrives at a given site, establishment is based on the abundance of other species in the cell and the shade tolerance rank of the seeding species relative to the species occupying the cell. We compiled the vital attributes of each species (Table 1) based on previous studies and forest inventory data in the study area [29], [37], [38]. LANDIS records species age cohort presence/absence. It tracks cells (sites) where mature age cohort present and uses a decay function to simulate the probability of seed reaching the surrounding cells. Seed source is considered abundant if high proportions of cells in the landscape containing mature age cohorts.

**Table 1:** Longevity, shade tolerance, effective and max seeding distance for each simulated species.

Species	Longevity (years)	Shade tolerance (class)^a^	Effective seeding distance (m)^b^	Max seeding distance (m)^b^
Fir	200	5	20	100
Korean pine	300	4	50	200
Spruce	300	4	50	150
Larch	300	2	100	400
Oak	300	2	20	200
Mountain birch	200	1	100	300
White birch	150	1	200	4000

a Shade tolerance value (1–5). 1 =  least tolerant; 5 =  most tolerant.

b The effective seeding distance is the distance at which seed has the highest probability (e.g., *P* > 0.95) of reaching a site. The maximum seeding distance is that distance beyond which a seed has near zero probability (e.g., *P*<0.001) of reaching. Seed-dispersal probability (*P*) between the effective (*ED*) and maximum seeding distance (*MD*) follows a negative exponential distribution [51].

### 2.3 Design of simulation scenarios

**Figure 3:**
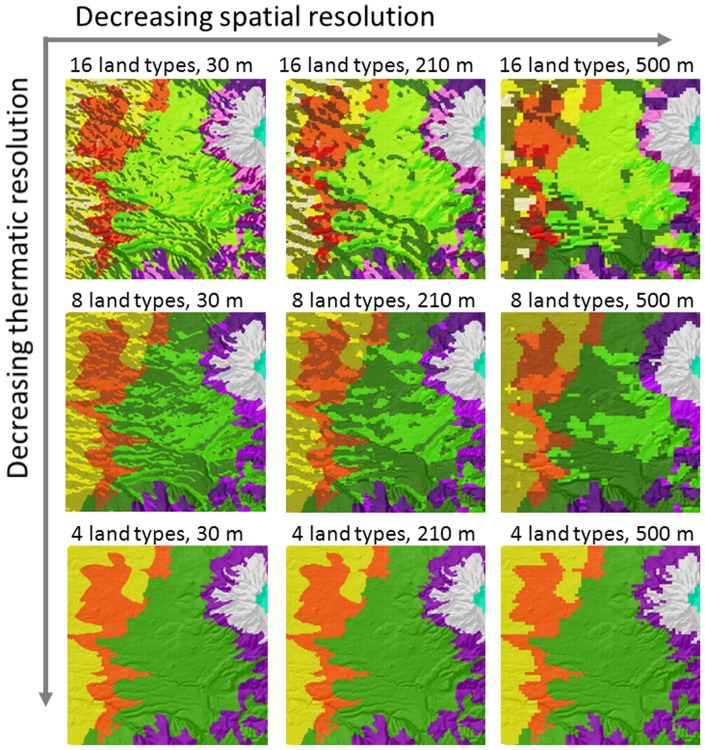
Changes in spatial configuration of land type maps with thematic and spatial resolutions. Each large square is a part of study area and different colours represent different land types within study area.

To investigate the effects of thematic and spatial resolutions in characterizing environmental heterogeneity on forest landscape predictions, we designed a series of scenarios with different combinations of thematic and spatial resolution for land type maps that characterized the environmental heterogeneity of the study area (Fig. 3). Each land type map has a set of SEPs for one species (Table 2). Each scenario has the same forest composition map to keep the initial species distribution consistent.

**Table 2:** Species-specific establishment probabilities for three land type maps under warming climate.

Land type		Species						
Elevation^a^	Aspect^b^	Fir	Korean pine	Spruce	Larch	Oak	Mountain birch	White birch
**4 land types**								
HWF	-	0.000	0.459	0.000	0.000	0.791	0.000	0.622
MKH	-	0.010	0.553	0.013	0.193	0.726	0.000	0.436
SFF	-	0.684	0.141	0.729	0.382	0.016	0.164	0.081
SAF	-	0.246	0.000	0.267	0.256	0.000	0.307	0.000
**8 land types**								
HWF	Sunny	0.000	0.459	0.000	0.000	0.870	0.000	0.684
	Shady	0.000	0.556	0.000	0.000	0.870	0.000	0.684
MKH	Sunny	0.009	0.498	0.012	0.191	0.718	0.000	0.431
	Shady	0.012	0.669	0.016	0.212	0.798	0.000	0.479
SFF	Sunny	0.616	0.127	0.656	0.378	0.015	0.148	0.072
	Shady	0.828	0.171	0.882	0.420	0.017	0.181	0.089
SAF	Sunny	0.234	0.000	0.254	0.255	0.000	0.291	0.000
	Shady	0.298	0.000	0.294	0.282	0.000	0.338	0.000
**16 land types**								
HWF	North	0.000	0.556	0.000	0.000	0.870	0.000	0.684
	South	0.000	0.459	0.000	0.000	0.870	0.000	0.684
	East	0.000	0.436	0.000	0.000	0.789	0.000	0.620
	West	0.000	0.506	0.000	0.000	0.830	0.000	0.653
MKH	North	0.012	0.669	0.016	0.212	0.798	0.000	0.479
	South	0.009	0.498	0.012	0.191	0.718	0.000	0.431
	East	0.010	0.553	0.013	0.202	0.762	0.000	0.457
	West	0.011	0.610	0.015	0.212	0.762	0.000	0.457
SFF	North	0.828	0.171	0.882	0.420	0.017	0.181	0.089
	South	0.616	0.127	0.656	0.378	0.015	0.148	0.072
	East	0.650	0.134	0.693	0.381	0.015	0.156	0.077
	West	0.791	0.163	0.842	0.441	0.017	0.181	0.089
SAF	North	0.298	0.000	0.294	0.282	0.000	0.338	0.000
	South	0.234	0.000	0.254	0.255	0.000	0.291	0.000
	East	0.222	0.000	0.241	0.230	0.000	0.276	0.000
	West	0.272	0.000	0.295	0.269	0.000	0.322	0.000

a HWF: the hardwood forest zone; MKH: the mixed Korean pine hardwood forest zone; SFF: the spruce–fir forest zone; SAF: the subalpine forest zone;

b: Sunny: the sunny slope; Shady: the shady slope; North: the north slope; South: the south slope; East: the east slope; West: the west slope.

Thematic resolution of land type map included three levels (4, 8, and 16 land types), which were characterized by environmental variables (elevation and aspect) (see Section study area for explanations of these three thematic resolutions). We used the maximum number of land types to represent a high thematic resolution, whereas fewer land types represented lower thematic resolutions.

We included six spatial resolutions: 30 m, 90 m, 150 m, 210 m, 250 m and 500 m. This wide range of spatial resolutions is sufficient for most studies involving common remotely sensed data (e.g., Landsat TM/ETM^+^ and MODIS). As the grain size increased, data was aggregated following the majority rule, which is the most commonly used method for aggregating categorical data in ecology and remote sensing [15], [39]. In total there were 18 scenarios (three levels of thematic resolution × six levels of spatial resolution). In order to obtain the identical data input under different spatial resolutions, we resampled 500 m, 250 m, 210 m, 150 m, and 90 m resolution forest composition map and land type map to 30 m. This ensured that the simulated differences were not due to different input data.

### 2.4 Model simulation

We used LANDIS 6.0 (landis.missouri.edu), an expanded version of LANDIS 4.0, to simulate our study area from 1990 to 2190 (200 years) at 5-year time steps. We simulated seven of the most common tree species within our study area: Korean pine, spruce, fir, birch, larch, mountain birch, and oak. These species cover near 90% of the forest in our study area. We completed five replications starting with the same input parameters, with the exception of a random seed number used to account for the effects of stochastic components, such as seed dispersal and seedling establishment. Disturbance such as forest harvesting, fire, and wind were not simulated because our objective was to examine the natural successional trajectories of the most common tree species.

### 2.5 Data analysis

We used LandStat 6.0, an ancillary program of LANDIS 6.0, to process the simulation results. These simulation results were summarized as species abundance (the number of pixels in which a species occurs divided by the total number of pixels).

Initially we compared the differences in mean abundance of the entire simulated period among different levels of thematic resolution and different levels of spatial resolution, respectively, by one-way ANOVA (data satisfy the fundamental conditions of ANOVA: independent random samples, normality and homogeneity of variances in the residuals). Significant differences indicated that the choice of thematic/spatial resolutions affected landscape-scale predictions of tree species distribution. We then examined whether the differences among different thematic resolutions varied at different levels of spatial resolutions and whether the differences among different spatial resolutions varied at different levels of thematic resolutions. The variation revealed the interaction of thematic and spatial resolutions.

To investigate the relative importance of thematic and spatial resolutions on forest landscape predictions, we conducted a two-factor univariate analysis using General Linear Model (SPSS 16.0). The dependent variables (abundance of simulated species) were tested for normality and homogeneity of variances in the residuals. Two-factor independent variables (thematic and spatial resolution) were both fixed factors. Type III sums of squares derived from the univariate analysis were used to quantify the relative importance of thematic resolution, spatial resolution, and their interaction with forest landscape predictions [29], [40]. Higher type III sums of square values indicated larger contributions to the predicted species abundance. The actual type III sums of square values of thematic resolution, spatial resolution, and their interaction were comparable within one statistical model but not necessarily between two or more statistical models. Therefore, we transformed the actual type III sums of square values into proportions for comparing the differences of the relative importance of thematic and spatial resolutions, and their interaction among simulated species [40].

After the above analysis, we found that four species, Korean pine, fir, birch and larch captured the response patterns of all seven species. Thus, we only presented the results of these four species.

## Results

### 3.1 Relative importance of thematic and spatial resolution

**Figure 4:**
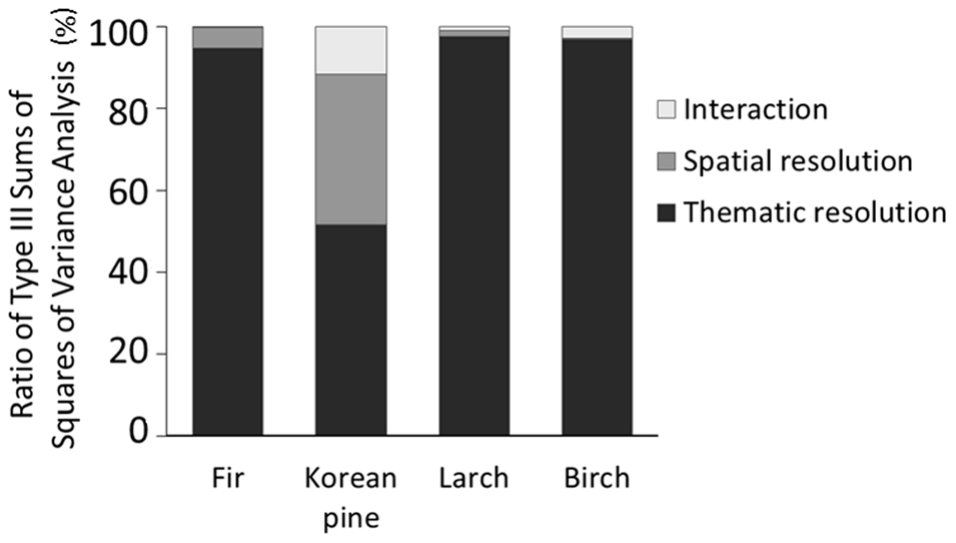
The relative importance of thematic and spatial resolution, and their interaction. Ratio of the type III sums of square values of a fixed model (both thematic and spatial resolution are fixed factors) corresponding with the relative importance of thematic and spatial resolution, and their interaction to species distribution prediction at the landscape scale.

For most species (e.g., fir, larch and white birch), the proportions of type III sums of square values for thematic resolution were obviously larger than those for spatial resolution and their interaction, indicating that the relative importance of thematic resolution effects on predictions of species distribution was far larger than spatial resolution. For example, for fir, the proportion of type III sums of square values of thematic resolution was near 95%, whereas the proportion of spatial resolution was only 5% (Fig. 4). By contrast, for Korean pine, the proportion of type III sums of square value for thematic resolution (52%) was slightly larger than that for spatial resolution (37%) (Fig. 4), indicating thematic resolution accounted for more than half of the changes in predictions of species distribution, while spatial resolution made up a nearly half contribution.

### 3.2 Changes of predicted species abundance with varying thematic resolutions

**Figure 5:**
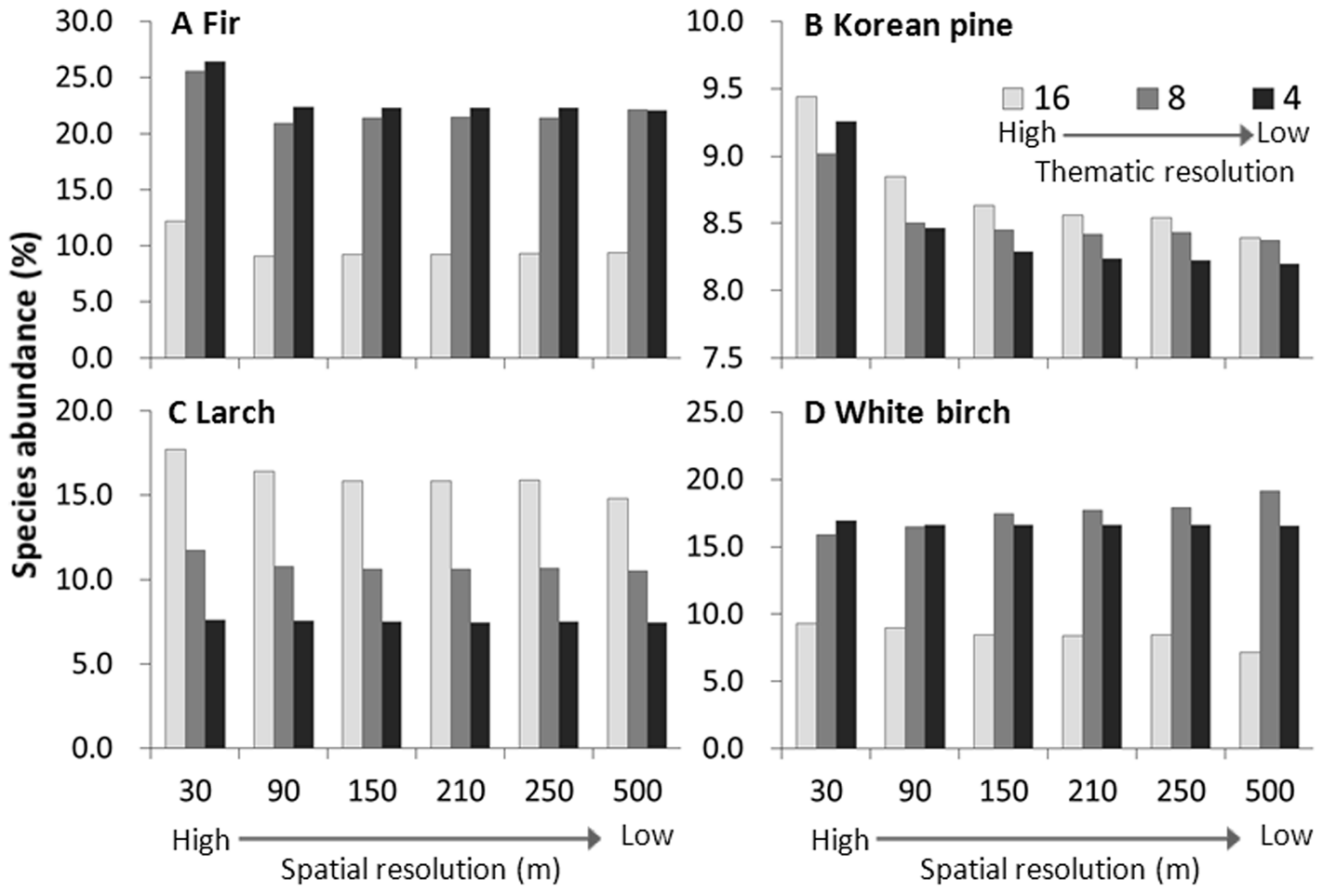
Mean abundance at different thematic and spatial resolutions. Mean abundance of some simulated tree species at different thematic resolutions (three levels: 4, 8 and 16 land types) and spatial resolutions (six levels: 30 m, 90 m, 150 m, 210 m, 250 m and 500 m).

Results of ANOVA showed significant that for all species simulated, significant differences (*p*<0.001) in abundance occurred among different levels of thematic resolution (Table 3). For Korean pine and larch, mean abundance decreased as thematic resolution decreased. For example, under 30 m spatial resolution, mean abundance of larch from high to low thematic resolutions were 17.68, 11.70 and 7.62%, respectively; under 500 m spatial resolution, mean abundance from high to low thematic resolutions were 14.81, 10.49 and 7.45%, respectively (Fig. 5c).

**Table 3:** ANOVA results for species abundance.

		Fir	Korean pine	Spruce	Larch	Oak	Mountain birch	White birch
**Thematic resolution**								
4 land types	% mean	22.91	8.44	15.43	7.51	6.44	4.74	16.65
	SD	1.59	0.38	0.37	0.06	0.05	0.00	0.14
8 land types	% mean	22.14	8.16	21.44	10.81	10.51	7.06	17.42
	SD	1.58	0.11	0.66	0.41	0.94	0.07	1.07
16 land types	% mean	9.74	7.62	13.20	16.08	7.80	6.36	8.46
	SD	1.11	0.43	0.50	0.87	0.43	0.18	0.68
F-value		785.90	46.34	1993.38	1790.06	364.92	3313.26	1372.55
*p*		<0.001	<0.001	<0.001	<0.001	<0.001	<0.001	<0.001
**Spatial resolution**								
30 m	% mean	21.37	8.67	16.74	12.34	9.25	6.18	14.03
	SD	6.74	0.43	2.61	4.28	2.55	1.06	3.51
90 m	% mean	17.46	8.13	16.99	11.56	8.19	6.02	14.03
	SD	6.15	0.27	3.78	3.79	1.74	0.97	3.68
150 m	% mean	17.65	8.00	16.70	11.32	8.09	6.03	14.16
	SD	6.17	0.34	3.82	3.57	1.62	1.01	4.20
210 m	% mean	17.66	7.95	16.69	11.31	8.07	6.04	14.24
	SD	6.16	0.32	3.77	3.57	1.62	1.00	4.31
250 m	% mean	17.63	7.91	16.73	11.34	8.05	6.03	14.32
	SD	6.13	0.33	3.77	3.58	1.59	0.99	4.30
500 m	% mean	17.83	7.77	16.28	10.92	7.83	6.02	14.28
	SD	6.16	0.59	3.89	3.13	1.40	1.04	5.33
F-value		0.89	9.77	0.06	0.25	1.19	0.06	0.01
*p*		>0.05	<0.001	>0.05	>0.05	>0.05	>0.05	>0.05

By contrast, mean abundances of fir and white birch under high thematic resolution (16 land types) were less than those under low thematic resolution (4 land types). Mean abundance of fir at 30 m resolution under high thematic resolution was 12.18% lower than those under medium and low thematic resolution (25.54% and 26.40%, respectively) (Fig. 5a). A similar trend was also observed at 500 m spatial resolution for fir. Mean abundance of white birch under high thematic resolution (9.28%) was also lower than those under medium and low thematic resolution (15.86% and 16.95%, respectively) at 30 m spatial resolution (Fig. 5d). However, at 500 m resolution mean abundance under medium thematic resolution (19.14%) was higher than those under high and low thematic resolutions (16.54 and 7.16%, respectively).

For Korean pine, larch and fir, when spatial resolutions were high, the differences of mean abundance among different thematic resolutions were higher than the differences under low spatial resolutions. For example, the differences of mean abundance of larch among different thematic resolutions decreased (from 10.06% to 7.36%) as spatial resolutions decreased. By contrast, for white birch, the differences of mean abundance among different thematic resolutions increased (from 7.67% to 9.38%) as spatial resolution decreased.

### 3.3 Changes of predicted species abundance with varying spatial resolutions

Results of ANOVA showed that there were no significant differences (P > 0.05) in abundance of the simulated species except Korean pine (*p*<0.001) among different levels of spatial resolution (Table 3). The mean abundance of most species decreased with decreasing spatial resolution (Fig. 5). For example, for larch, under high thematic resolution, mean abundances of larch from high to low spatial resolutions were 17.68% (30 m), 16.40% (90 m), 15.85% (150 m), 15.83% (210 m), 15.88% (250 m) and 14.81% (500 m), respectively; under low resolution, mean abundances from high to low spatial resolutions were from 7.62 to 7.45% (Fig. 5c). White birch has a similar trend as larch under high and low thematic resolution. However, mean abundance of white birch increased as spatial resolutions decreased under medium thematic resolution.

In addition, when thematic resolutions were high, the differences of mean abundance among different spatial resolutions were generally larger than the differences under low thematic resolutions.

## Discussion

Our results showed that thematic and spatial resolutions used in characterizing environmental heterogeneity have different effects on model predictions of abundance for all simulated tree species. The different responses to varying thematic and spatial resolutions from individual tree species may be due to species' ecological traits and interspecies competition as implemented in the LANDIS.

### 4.1 The effects of thematic resolution

Under high thematic resolutions, land types are classified and mapped in greater detail than those under low thematic resolutions. Increasing land type diversity led to the simulated diverse species responses to environments, which are characterized as species establishment probabilities (SEPs) in this study. Under low thematic resolutions, the detailed land types are combined into fewer classes and larger patches, and consequently, diverse species responses to environments (SEPs) are aggregated. Gain or loss of abundance with varying thematic resolution depends on the species' ecological traits and interspecies competition.

Results showed that for species having moderate dispersal distance (>50 m) and relatively abundant seed sources, abundance decreased as thematic resolution decreased. This is because the aggregation process reduces the SEPs for land types with relatively high suitable establishment conditions to an average value under which the species have low probabilities to establish. Moreover, under high thematic resolutions, once seedlings established, they begin to disperse more new seeds after the seedling matures in a few decades, and thus, abundance increases exponentially when seed sources and dispersal distance are not limiting [36]. Such a process may not occur when few seeds can established with the averaged SEPs under low thematic resolutions, resulting in lower species abundance [25]. This phenomenon was reflected by species simulated in our study, such as larch with moderate effective seeding distance (100 m) and abundant seed sources. The predicted abundance of larch increased exponentially on land types with high SEPs under high thematic resolution, which resulted in predicted abundance of larch under high thematic resolutions significantly higher than those under low thematic resolutions.

As thematic resolution decreased, the aggregation of SEPs corresponds to changes in ‘source/sink relationship’ [41]. In a source/sink landscape, relatively productive land types with high suitable establishment conditions (with high SEPs) can serve as sources, which disperse seeds to less productive land types (with low SEPs) called sinks [42], [43]. Species distribution is affected by the relative ratio of source and sink [44], [45]. As thematic resolution decreases, land types with high SEPs are averaged resulting a reduction of source. Consequently, species abundance decreases as thematic resolution decreases.

It may seem obvious that higher thematic resolutions result in higher species abundance. However, for a given landscape, an increase in the number of land types leads to a decrease in the amount and size of units for each land type [20]. This may produce an inverse effect of thematic resolution on predicted abundance for species with long seeding distances that need large areas or nearly contiguous habitat, as they may disappear from land types in which their required area is inadequate [26], [46]. This could lead to a decrease in abundance under high thematic resolution, i.e. a peak in abundance under intermediate thematic resolution. This phenomenon was reflected by species simulated in our study, such as white birch with a long dispersal distance (e.g., 200–4000 m). Predicted abundance of white birch under intermediate thematic resolutions was larger than those under high and low thematic resolutions.

Results also showed that for the most shade-tolerant species (e.g., fir), which was more competitive than the other species, abundance increased as thematic resolution decreased. This is because high shade tolerance allows the species to compete with shade intolerant species on land types with relatively low establishment probabilities under low thematic resolutions. Moreover, when thematic resolution is high, seed dispersal between units of the same land type is reduced, because of reduced cross-unit synchrony in the landscape dynamic [47], [48], whereas decreasing thematic resolution increases seed dispersal between units of the same land type. This could lead to an increase in abundance under low thematic resolution.

### 4.2 The effects of spatial resolution

In contrast to thematic resolution, a decrease in spatial resolution does not decrease compositional heterogeneity. Rather, decreasing spatial resolution may decrease the number of land type units and increase the area of individual unit and connectivity between units of the same land type, thus decreasing configurational heterogeneity [15], [22], [39].

**Figure 6:**
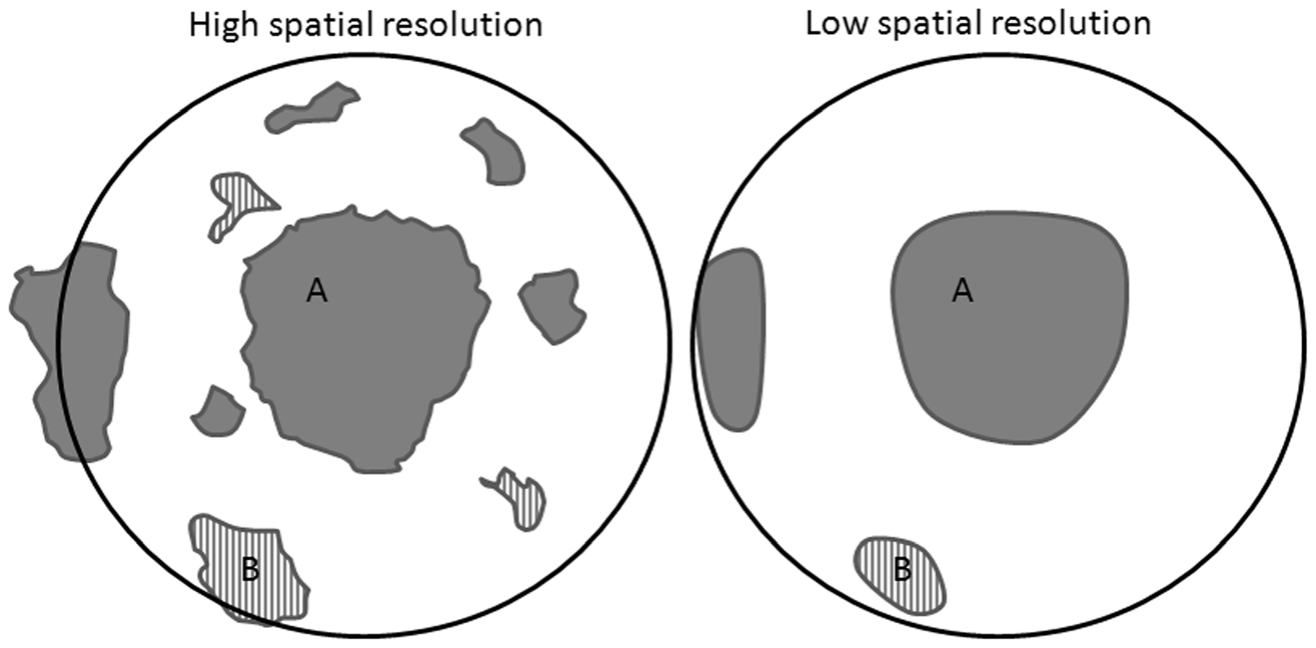
Landscape supplementation. A species could supplement its resource intake from land type A or similar resources from land type B. High spatial resolution supports more landscape supplementation than low spatial resolution as long as these units of land type are within seeding distance (dark oval).

Results showed that abundance increased as spatial resolution increased when seed sources and dispersal distance were not limiting. This is because increases in configurational heterogeneity may increase ‘landscape supplementation’, in which species could supplement their resource intake (available habitats) from nearby habitats (land types with high SEPs), or by using a substitutable resource in nearby similar land types (habitats) [41]. High spatial resolution results in more land type units than those under low spatial resolution, which increases landscape supplementation (Fig. 6). In addition, more complex shapes of units of land types increase interspersion/juxtaposition and the length of boundaries between potentially supplemental resources, and consequently increase landscape supplementation [26], [49]. This increase leads to a high species relative abundance under high spatial resolutions. This phenomenon was reflected by species simulated in our study, such as Korean pine, spruce, fir, and larch, which have moderate dispersal distance and relatively abundant seed sources. While concepts such as landscape supplementation were developed by survey of animal populations [50], it is not restricted to this context. Our results suggest that it is also applied to corroborate the effect of spatial configuration on predictions of species distribution.

### 4.3 The relative contributions

We found that for all simulated species the effects of thematic resolution on predictions of landscape-scale species distribution were larger than the effects of spatial resolution. This is probably at least partly because increasing thematic resolution alters the relative ratios of source and sink by increasing compositional heterogeneity. Meanwhile, thematic resolution may increase landscape supplementation by increasing configurational heterogeneity, based on the same arguments for increasing configurational heterogeneity as spatial resolution increases [26], [41].

Our results also showed that spatial resolution had an influence on the effects of thematic resolution on species distribution prediction. When spatial resolution is low, increasing thematic resolution will increase the unit number of neighbouring land types very little and will have a small influence on landscape supplementation, resulting in a small difference in species abundance among different thematic resolutions. In contrast, increasing the thematic resolution when spatial resolution is high will increase the unit number of surrounding land types and thus will increase landscape supplementation, resulting in a relative large difference in predicted distribution among different thematic resolutions.

## Conclusion

Results showed that both thematic and spatial resolutions in characterizing environmental heterogeneity affected model-based predictions of species distribution, but thematic resolution had a stronger effect on predictions than spatial resolution. Species abundance increased as thematic resolution increased for species having moderate dispersal distance under the precondition of relatively abundant seed sources. However, an inverse effect of thematic resolution on predicted abundance may be produced for species with long seeding distance or shade-tolerant species. In addition, species abundance increased as spatial resolution increased, provided that seed sources and dispersal distance were not limiting, due to increases in configurational heterogeneity resulted from increasing spatial resolution, may increase ‘landscape supplementation’.
